# Supplementary material for: Are carbon emissions trading and green financial instruments synergistic? -Comprehensive quantitative research based on content analysis
Source: PLoS One. 2024 Mar 7;19(3):e0298601. doi: 10.1371/journal.pone.0298601 (PMC10919601; doi:10.1371/journal.pone.0298601)
Supplement: S1 Data — (ZIP) [file pone.0298601.s001.zip › Data/地级市Z_F工作报告.zip!/╕╩╦α/╬Σ═■╩╨/└┤╫╘╣1⁄2╓┌║┼í╛┬φ┐╦╩2╛▌═°í┐_2015.docx]

各位代表：

现在，我代表市人民政府，向大会作工作报告，请予审议，并请各位政协委员和列席人员提出意见。

2014年工作回顾

过去一年，面对复杂多变的经济形势和艰巨繁重的改革发展稳定任务，在省委、省政府和市委的坚强领导下，我们认真贯彻党的十八大和十八届三中、四中全会精神，全面落实省委“两手抓两手硬、双促进双落实”的重大战略部署，牢牢把握稳中求进的总基调，强化“以发展凝聚人心、用项目衡量政绩，无中生有抓项目、有中生优增效益，严格标准选干部、不拘一格用人才”的发展理念，全力实施工业强市、城乡融合和生态立市战略，全面落实稳增长、促改革、调结构、惠民生、防风险的各项措施，改革创新，真抓实干，全市呈现出经济较快增长、结构优化升级、改革有序推进、人民生活改善、社会事业发展、生态环境好转、社会大局稳定的良好局面。实现生产总值405.97亿元，增长9.1%；全社会固定资产投资836.2亿元，增长22.11%，其中规模以上666.37亿元，增长22.17%；全部工业增加值119.72亿元，增长11.4%，其中规模以上97.05亿元，增长10.8%；社会消费品零售总额131.7亿元，增长12.7%；公共财政预算收入22.18亿元，增长20.87%；城镇居民人均可支配收入19036元，增长9.6%，农民人均纯收入7834元，增长12.5%；居民消费价格指数累计达101.6%。

需要说明的是，国家统计局根据宏观经济政策调整变化，对相关统计标准作了调整。省政府依照这个情况，提请省人大对部分指标作了调整。为此，经市委同意并报请市人大常委会批准，对我市年初确定的相关指标也作了相应调整。

一年来，主要做了以下九个方面工作：

一、狠抓项目建设不动摇，经济社会发展后劲增强。

围绕实施“3341”项目工程，着力营造“能赚钱、不受气、条件好”的投资环境。全年共实施500万元以上项目955项，其中亿元以上310项。实施招商引资项目760项，落实到位资金810亿元，增长37%。金色大道、荣生大道、天颐大道建成通车，民勤县城至红沙岗一级公路、天祝至青海互助二级公路建设进展顺利，干武铁路增建二线开工建设，北仙高速公路奠基。新建改建农村公路1131.6公里。天祝县南阳山片“下山入川”生态移民小康供水工程建成通水，杂木河毛藏寺水库枢纽工程加快推进。

二、着力培育壮大主导产业，经济结构不断优化。

三次产业结构由2009年的27.7∶38.3∶34调整为23.5∶42.7∶33.8。

着力构建新型工业体系。实施千万元以上工业项目267项，其中亿元以上141项，完成工业投资336亿元，增长28.4%。新增规模以上企业44户，居全省第2位。京奥港30万吨高档饮品等一批重点项目竣工投产。鑫淼60万吨电石、200万吨新型干法水泥等一批重点项目加快推进。荣华30万吨鲜奶制品、1.5万吨婴儿奶粉等一批重点项目开工建设。新建成风光电项目1007兆瓦，累计达到2150.5兆瓦。武威热电联产项目核准并启动建设。组建了甘肃省风电装备制造全产业链联盟。加快推进电网建设，雷台、红沙岗两个330千伏输变电工程建成运营。大力改善园区道路、供排水等基础条件，完成投资18亿元。黄羊工业园区国家级循环化改造项目进展顺利，“古浪鑫淼模式”和“黄羊河模式”入选全省循环经济发展典型案例。积极推进工业节水和节能减排，单位工业增加值用水量下降3%，关闭年产3万吨以下小煤矿19处，节能减排任务全面完成。实施城市光网建设、行业信息化应用等项目，促进了信息化和工业化融合发展。

大力发展现代农业。强力推进主体生产模式建设，新建设施农牧业19.2万亩、特色林果基地33万亩。新建养殖场（小区）358个，新发展规模养殖户1.22万户。全市牛、羊、猪存栏量分别达到83.5、593.2、153.4万头（只），肉蛋奶总产量达到30.8万吨。推广旱作农业72.2万亩、高效农田节水技术252.4万亩。加大标准化技术规范体系建设，制定和推行番茄、辣椒、肉羊、皇冠梨等31个出口农产品生产技术规程。加快发展“贮藏加工+运输销售”现代营销模式，大力拓展国内外市场，设施农产品外销量占总产量的70%以上。

积极发展现代服务业。黄羊公铁联运物流中心、莫高国际生态酒堡等项目基本建成，红星时代广场开工建设。新建续建万嘉国际广场等市场15个，升级改造县乡农贸市场7个、农家店和商品配送中心225个，建成黄羊河等大宗农产品冷藏储备项目4个。武威至兰州城际直达旅客列车开行。粮食购销量达到153.8万吨，市、县区级储备粮规模达到4.4万吨。成功举办“中国•河西走廊第四届有机葡萄美酒节”。 加快华夏文明传承创新区武威项目区建设，天马文化产业园摩天轮单体项目建成投运，天梯山生态文化旅游区、天祝冰沟河生态文化旅游景区开发等项目进展顺利，文庙修缮保护一期工程完工。全年共接待游客631.2万人，增长23.6%；实现旅游业综合收入31.75亿元，增长27.35%。

三、深入推进城乡融合，城镇化进程明显加快。

加快城镇基础设施建设。建成城乡融合发展核心区干支路网371.5公里。凉州新城区建成道路6条，滨河东路完成路基工程，天马湖景观治理二期工程实现蓄水，污水再生利用项目完成主体工程，新关村—荣华老厂区棚户区改造项目启动实施。城区建成人行过街天桥3座。凉州城区实现地表水供水全覆盖，居民全部用上了优质天然矿泉水。解决了20万农村人口安全饮水问题。民勤县全省农村危房改造示范县和美丽乡村建设试点县规划、建设同步推进。

优化城乡空间布局。民勤县和凉州区黄羊镇、古浪县黄羊川镇新型城镇化试点启动实施。民勤县域核心区总体规划和县城总体规划正在修订，天祝县域核心区总体规划修订完成。

有序推进农村转移人口市民化。建立城乡统一户籍制度，完成农业转移人口居民户口登记2.11万户、7.37万人。全市城镇化率达到34.34%，提高2个百分点。

四、坚持以节水造林治沙防污为重点，生态文明建设取得新成效。

扎实推进石羊河流域后期治理。蔡旗断面过水量达到3.188亿立方米，民勤盆地地下水开采量控制在8506万立方米，青土湖形成水面22平方公里，地下水埋深小于3米的旱区湿地约106平方公里，提前6年实现国家规划确定的2020年生态治理目标。深化水权水价改革，严格落实总量控制和定额管理制度，运用价格杠杆促进了节约用水。

加大防沙治沙和造林绿化力度。扎实推进石羊河流域防沙治沙及生态恢复项目、天保工程、中央财政造林补贴项目和省级财政防沙治沙项目、民勤沙化土地封禁保护补助试点项目。推广应用防沙治沙技术，加快建设防沙治沙示范区。打造通道绿化和园区绿化精品示范工程。坚持机关干部职工义务压沙造林制度常态化，扎实推进全民义务植树活动，开展大规模秋冬季压沙造林活动。完成人工造林63.2万亩，封育36.6万亩。

大力发展工业治沙和绿色养殖治沙。创新沙漠治理新思路，利用沙漠光热资源优势，发展沙漠光伏发电基地。以荣华绿洲现代农业产业园、长庆现代农业产业园为重点，规模化养殖、集约化经营，在沙漠沿线建成一批养殖小区。推进治沙生态林承包经营机制，将国有治沙生态林承包到户，发展沙生药用植物为主的沙产业。承包到户面积30万亩，累计达到180.9万亩。

坚决治理环境污染。落实最严格的源头保护制度和节能减排目标责任制，实施大气污染防治行动和清洁水行动计划，全市空气质量优良天数329天，地表水水质达标率100%。

五、加快发展社会事业，公共服务能力不断提升。

优先发展教育事业。新改建校舍16.7万平方米，消除D级危房8.83万平方米，建成幼儿园20所，实现全市乡镇中心幼儿园全覆盖。民勤县顺利通过国家“县域义务教育发展基本均衡县”评估认定。高考录取率达到81.7%。武威职业学院一期扩建项目有序推进，兰州交通大学新能源与动力工程学院、甘肃威龙葡萄酒专修学院在校生分别达到871人和630人。凉州职业中专创建国家中等职业教育改革发展示范校顺利通过省级评估验收。

健全完善医药卫生服务体系。深化医药卫生体制改革，基本药物制度覆盖基层，县级公立医院改革试点稳步实施。重离子治疗肿瘤中心暨荣华颐养园、市人民医院整体搬迁一期工程完成主体，市中医院综合服务楼项目建设进展顺利。新建改建妇幼保健院2 所、急救中心2个、乡镇卫生院29个、标准化村卫生室227个。落实单独两孩政策，人口自然增长率控制在7‰以内。

大力发展文化体育事业。公共文化服务体系不断完善，市级图书馆、美术馆实现了免费开放。市体育馆、影剧院、博物馆建设顺利推进。大力开展全民健身活动，新建乡镇及社区文化体育健身中心9个。

协调发展其他社会事业。天祝“两个共同”示范县建设深入推进，少数民族地区发展步伐加快，民族和睦、宗教和顺。国防动员和“双拥”工作得到加强。统计、气象、人防、消防、老龄、外事侨务、防震减灾、档案管理、地方志、妇女儿童、残疾人和慈善事业等工作都取得了新的成绩。

六、认真解决群众切身利益问题，民生改善持续加强。

着力保基本、兜底线、惠民生、促稳定，省列23件、市列10件实事全部完成。

加强就业创业工作。全市城镇新增就业3.88万人，应届普通高校毕业生就业率达到90%，城镇登记失业率控制在3.5%以内。

提升社会保障水平。对全市城乡低保对象进行全面复核，新纳入1.01万人。城市低保标准由294元提高到339元，农村低保标准由1907元提高到2193元，农村五保供养标准由2700元提高到3218元。新型农村合作医疗和城镇居民基本医疗保险政府补助标准由人均280元提高到320元。为6200名特困残疾人发放生活补贴744万元。发放救灾及社会救助资金6.2亿元。市第二社会福利院、市幸业养老中心等工程建设进展顺利，在城市和农村分别建成社区老年人日间照料中心7个和83个。

改善城乡居民住房条件。基本建成保障性住房41573套，分配入住17993套。新建保障性住房42956套全部开工。实施农村危房改造、新房建设5.17万户，新建续建新农宅集中示范点69个。

推进 “1236”扶贫攻坚行动。落实财政专项扶贫资金4.52亿元，48个村实现整体脱贫，预计减贫12.65万人。全力实施“下山入川”工程，新建天祝南阳山片松山滩、古浪黄花滩生态移民区圆梦新村等移民安置点13个，完成“下山入川”移民2.27万人。荣华绿洲移民社区开工建设。

加强社会治理工作。完善矛盾纠纷排查化解研判制度，矛盾纠纷化解率达到99%。加大食品药品安全监管力度，严厉打击食品药品安全违法犯罪行为，保证了人民群众饮食用药安全。严格落实安全生产责任制，深入开展打非治违和隐患排查治理专项行动，未发生较大以上安全生产事故。

七、狠抓全面深化改革措施落实，经济社会发展动力明显增强。

全面落实市委“改革21条”，积极稳妥推进重点领域和关键环节改革。

认真落实商事制度改革措施。取消最低注册资本限制，将企业年检制度改为年度报告制度，全市新增各类市场主体1.58万户、注册资本金318.92亿元，分别增长11.5%、81.9%，市场主体达到9.61万户。

深入推进财税改革。全面推行部门预算和国库集中收付改革，加强财政资金拨付时效监管，强化“三公”经费管控，公务卡制度改革顺利推开。制定市级党政机关差旅费、会议费、培训费管理办法等制度。推进政府采购和公共资源交易改革，国有资产处置管理进一步加强。“营改增”税收改革试点有序推进。健全完善融资偿债机制和债务预警机制，政府性债务管理进一步规范。

加大金融改革发展力度。凉州区农信社成功改制为武威农商银行，甘肃银行在三县设立支行，小额贷款公司达到16家，融资性担保机构达到8家。成功发行武威企业债券13亿元和武威私募债券10亿元，国开行甘肃分行棚户区改造22.87亿元贷款计划下达，世行贷款1亿美元建设城乡融合发展核心区基础设施、德促贷款6700万欧元建设城西集中供热项目加快实施。积极推进农村产权抵押贷款模式，凉州区组建农村产权交易中心。继续扩大惠农贷款规模和覆盖面，全年发放“双业”贷款5.85万户、76.9亿元，“双联”贷款1.82万户、14.45亿元，信贷资金占设施农牧业投资的75%左右。至12月末，全市金融机构存款余额704.58亿元、增长16.4%，贷款余额527.95亿元、增长32.26%，存贷比74.93%。

加快科技创新体系建设步伐。甘肃武威国家农业科技园区挂牌启动，中科院西北高原生物所武威绿洲现代农业科学研究试验站落成揭牌。甘肃省酿酒葡萄苗木快繁工程技术研究中心立项建设，省级工程技术研究中心达到6家，省级企业技术中心达到15家。“武威酿造葡萄”和“民勤羊肉”荣获国家农产品地理标志登记认证。民勤县被列为国家知识产权强县工程试点县。大力推广甜高粱、山东黑牛等新技术、新品种，全市种植甜高粱达到12.42万亩。

深入推进农村改革。大力培育多元化农业生产经营主体，农民专业合作社达6137家，入社成员6.93万户，占农户总数的19%。流转农村家庭承包耕地79.5万亩，占家庭承包经营耕地面积的22%，涉及农户12.84万户，占农户总数的35%。林权确权颁证工作全面推开。

坚持管好用活土地资源。全市供应建设用地4.51万亩，保障了重点产业、重大项目和民生工程用地。实施“代建”“配建”基础设施、公益工程和安置住宅新模式。建立投资、用地协调机制，土地储备融资到位资金5.75亿元。落实土地整治项目19个，争取到位资金1.6亿元。集体土地所有权确权颁证全部完成。农村宅基地和建设用地使用权外业调查完成率分别达到97.9%、99.26%。办理抵押登记438宗，企业融资47.9亿元。

着力打造“丝绸之路经济带”武威黄金节点。甘肃首个海关特殊监管区—武威保税物流中心当年获批、当年建成运营，甘肃首列中欧国际货运班列“天马号”顺利开通。规划建设甘肃国际陆港，陆港发展战略规划及中心区控制性详细规划加快编制。山东天成食品集团年产1.8万吨宠物食品生产销售及进出口贸易一体化等项目成功入驻。引进建设美国温德克通航飞机、德国农业大型机械制造、美国月色美地葡萄酒堡及葡萄基地等一批国外投资项目。

八、加快转变政府职能，服务质量和效率显著提升。

扎实开展党的群众路线教育实践活动，“四风”问题得到有效遏制，群众反映强烈的一批突出问题得到解决。认真落实中央八项规定和省委、市委“双十条” 规定精神，市级会议减少35.2%，文件简报减少2.9%，“三公”经费支出下降26.4%。清理清退公务用车41辆，清理腾退办公用房3.55万平方米。深入实施效能风暴行动，突出整治不作为慢作为，对299个单位和239名责任人进行了严肃问责。大力推进简政放权，承接省级取消调整和下放行政审批项目85项，承接中央在甘单位取消行政审批项目7项。市级行政审批项目由295项精简为203项，精简92项。提升武威市行政权力网上公开透明运行系统运行水平，各类行政审批和管理服务事项全部纳入系统办理，实行电子监察，共受理行政审批和管理服务事项16.2万件，办结率99.5%。统筹推进事业单位分类改革。严格规范机构编制管理，消化超编人员389名，清理清退吃空饷人员51人。自觉接受人大法律监督和工作监督、政协民主监督，认真办理人大代表议案、意见建议和政协委员提案，政府公信力不断提高。

九、认真履行主体责任，政府系统党风廉政建设切实加强。

严格落实党风廉政建设主体责任，坚持把党风廉政建设与经济社会发展同部署、同落实、同检查、同考核。加强廉政教育，落实约谈制度，对政府系统18名县级干部进行诫勉或提醒谈话，对失职渎职造成不良影响的46名责任人实施了责任追究。强化廉政风险防控，扎紧制度“笼子”，实行“一把手”不具体分管人事、财务、行政审批、工程招投标等制度。切实加强廉政监管，加大对城乡低保、保障性住房、土地出让收支、财政扶贫资金等重点领域的监督检查力度，共清理清退城乡低保对象13066人，查改管理不规范扶贫资金748万元，查处违规纳入农村危房改造和城镇保障性住房130人，查处落实惠民政策优亲厚友、以权谋私问题34起，查处了一批在征地拆迁、环境保护等方面损害群众利益的问题。

各位代表，回顾一年来的工作，全市各级干部和广大人民群众同心协力，真抓实干，做了大量的工作，取得了较好的成绩。这是省委、省政府和市委统揽全局、正确领导的结果，是市人大、市政协和社会各界监督支持的结果，是全市各族人民共同努力的结果。在此，我代表市人民政府，向全市各族人民，向各民主党派、工商联、人民团体和社会各界人士、离退休老同志，向驻武各部队、武警官兵、民兵预备役人员和中央、省属驻武各单位，以及所有关心支持武威改革发展的同志们、朋友们，表示崇高的敬意和诚挚的感谢！

在看到成绩的同时，我们也认识到，经济社会发展中还存在一些困难和问题。主要是：受宏观经济环境变化影响，经济下行压力加大，企业生产经营困难增多，主要经济指标增速回落；“改革21条”的落实力度不够大，一些深层次的矛盾和问题解决不到位；招商引资签约项目跟踪落实和服务不到位，部分项目建设进展缓慢；改善民生任务重、扶贫攻坚难度大，群众反映强烈的一些突出问题还未得到全面有效解决；作风建设长效机制还不健全，解决“为官不为”、惩治“贪假懒庸”、防止“四风”问题、提升干部能力还需要持续用力。对此，我们必须高度重视，认真研究，全力解决。

2015年主要工作

2015年是全面完成“十二五”规划的收官之年，是全面深化改革的关键之年，也是贯彻落实党的十八届四中全会精神、全面推进依法治国的开局之年。政府工作的总体要求是：全面贯彻党的十八大、十八届三中、四中全会和中央经济工作会议精神，认真落实省委十二届九次全委会议暨全省经济工作会议和市委三届八次全委会议暨全市经济工作会议部署，协调推进全面建成小康社会、全面深化改革、全面依法治市、全面从严治党，强化“以党建统揽全局、用发展凝聚人心，无中生有抓项目、有中生优增效益，严格标准选干部、不拘一格用人才”的理念，全力实施工业强市、城乡融合、生态立市战略。坚持以经济建设为中心，主动适应经济发展新常态，牢牢把握稳中求进总基调，提高经济发展质量和效益，坚持经济发展与环境、资源、安全相协调，提高质量、效益与增加总量并重，强化发展基础，壮大经济实力。以改革促发展、促转方式调结构。坚持经济建设与社会事业发展同步、与持续增加城乡居民收入并举，着力改善民生。狠抓基层党组织建设，加强党风廉政建设和反腐败斗争。确保深化改革顺利推进、经济持续健康发展、依法治市全面推进、社会大局和谐稳定。

主要预期目标是：生产总值增长9%左右；工业增加值增长11%左右；社会消费品零售总额增长13%左右；全社会固定资产投资增长18%左右，其中规模以上增长15%左右；全部招商引资到位资金增长16%左右，其中省外到位资金增长15%左右；公共财政预算收入增长20%左右；城镇居民人均可支配收入增长10%左右，农民人均纯收入增长12%左右；居民消费价格涨幅控制在3%以内；城镇登记失业率控制在4%以内；人口自然增长率控制在7‰以内；城镇化率提高2个百分点；单位生产总值能耗和主要污染物排放量控制在省政府下达的目标内。

围绕上述目标，重点抓好九个方面的工作：

一、加大结构调整力度，加快转变经济发展方式。

主动适应经济新常态，加快调结构、转方式、提质量、增效益，着力发展战略性新兴产业。

突出抓好新兴工业经济。要在加强和改善现有企业管理，用高新技术改造提升传统产业的同时，坚持引项目、找矿藏、建园区、上规模、创品牌、增效益，着力构建新型工业体系。培育发展产业链经济，打造风光电装备制造、核技术民用装备制造、碳化硅新材料、飞机汽车制造、电石、制药、包装、物流、农产品精深加工、液体经济、甜高粱、现代畜牧业等产业联盟和产业集群。加大园区布局调整力度，按照每个园区有主导产业、龙头骨干企业的要求，加快荣华高新产业园、新能源与装备制造产业园等10个园区规划建设，优化产业布局。加大园区基础设施建设力度，完成投资20亿元。推进中心城区工业企业“出城入园”。加快发展新能源产业，争取将天祝松山滩百万千瓦级风电项目列入全省风电项目核准计划并开工建设。加大与甘电投集团、中电投公司的衔接力度，加快甘电投武威2×350兆瓦热电联产项目建设进度，力争2016年建成运营。积极推进中电投武威电厂2×1000兆瓦火电工程前期工作，争取早日开工建设。抓好重点骨干项目建设，力争新开工千万元以上项目130项，建成120项。围绕培育“百亿元企业”，全力抓好鑫淼电石及深加工产业链、荣华30万吨鲜奶制品、1.5万吨婴儿奶粉、京奥港50万吨高档饮品等项目建设。强力推动建成项目达产增效，2014年建成的120个项目要力争实现产值66亿元、增加值14亿元。强化工业经济运行调度服务，深入推进重点项目、企业互保共建和互为市场工作。新增规模以上工业企业20户以上。加快9个省级循环经济试点示范园区和14户试点企业建设，组织实施一批工业循环经济重点项目。

大力发展现代农业。坚定不移推进主体生产模式，狠抓设施农牧业提质增效。加大新技术、新品种、新装备引进和试验示范推广力度，消除闲置棚，实现棚均收入2万元以上。新建特色林果基地30万亩，累计达到150万亩以上。推广高效农田节水技术250万亩、旱作农业70万亩、甜高粱20万亩。扶持发展獭兔产业，加强引种、繁育、饲料、防疫等环节的技术指导，积极引进兔肉加工项目。支持荣华绿洲和长庆现代农业产业园建设。强力推行出口农产品标准化生产技术规程，在甘肃国际陆港规划布局范围及其它日光温室连片示范点、养殖场（小区）和连片特色林果示范基地全面推行标准化生产技术，建成出口农产品生产基地50万亩，辐射带动面积100万亩。农畜产品专业批发市场全部实行市场准入准出制度。加大产品产地认证力度，争取新认证“三品一标”农产品7个以上。加快发展“贮藏加工＋运输销售”现代营销模式，大力发展专业协会、专业合作社，引进农产品深加工和销售企业，培育贮藏、加工、包装、运销和出口产业链。加大动植物疫病防控工作力度，确保不发生重大疫情和病虫灾害。深入推进“双联富民”与扶贫攻坚行动深度融合，实施精准扶贫。加快贫困片区主体生产模式发展，贫困户新建设施农牧业2.8万亩，实现户均2亩棚目标。发展特色林果业5万亩，实现人均1亩经济林目标。大力实施“下山入川”工程，全面实现五年规划目标。加快推进黄花滩、松山滩、荣华绿洲产业园等移民区产业开发、基础设施和公共服务设施建设，加强移民生产生活服务、社会保障、治安管理。做好移出地生态保护、留居地村社农户的整合建设。力争121个贫困村整体脱贫，减贫12.3万人。

提升现代服务业发展水平。加快城乡市场建设，不断增强消费拉动力。抓好黄羊众远公铁物流中心、万嘉国际广场、红星时代广场、昊天农产品交易市场暨仓储物流中心等重点物流项目建设。大力发展连锁超市、农家店、社区超市，构建城乡商业新网络。积极推进华夏文明传承创新区武威项目区建设，开工建设武威雷台文化产业园、武威创意文化产业园等项目，编制完成金色大道•马踏飞燕大景区规划和天祝民族风情、山水生态文化游规划，高标准谋划打造武威雷台、鸠摩罗什寺、文庙等历史文化景点。抓好葡萄酒特色文化游、天梯山历史文化游、祁连山风情生态文化游、天祝三峡文化游、冰沟河生态文化游等景区建设。办好“中国葡萄酒城葡萄美酒节”。大力发展信息产业，加强与阿里巴巴集团等电商合作，推进电子商务，建设淘宝网“特色中国•武威馆”，实施“武威特产网上行”促销工程。认真实施粮食收储供应保障工程，不断扩大放心粮油覆盖面。加快发展交通运输、金融保险、社区管理、健康养老等现代服务业。

二、强力推进项目建设和招商引资，不断扩大经济总量。

以“3341”项目工程为总抓手，加大项目谋划争取实施力度，全力推动转型跨越。着力营造“能赚钱、不受气、条件好”的投资环境，力争实施亿元以上项目300项左右，在建项目规模保持在1000亿元以上。创新招商引资方式，不断扩大招商引资成果。健全跟踪服务落实机制，切实提高项目履约率、资金到位率和开工投产率。规范招商引资统计工作，严格督查考核，防止虚报、重报招商引资资金行为。抓好项目落地和组织实施，建成民勤县城至红沙岗一级公路、天祝至青海互助二级公路，加快干武铁路增建二线、北仙高速公路武威段、天景二级公路、金荣沙漠生态公路建设进程，开工建设武威机场、世行城乡融合发展核心区基础设施项目、金色大道大靖至天景公路连接线。全面建成杂木河毛藏寺水库枢纽工程、石门河调蓄引水工程，开工建设红崖山水库加高扩建工程和甘肃国际陆港中心区、民勤红沙岗工业园区供排水工程，争取实施南营、西营、黄羊水库清淤工程。全力做好武威通航产业基地、兰张高铁等项目前期工作，力争启动实施。争取110千伏市政变电站、110千伏黄花滩变电站建成投运。

三、坚持城乡融合发展，加快新型城镇化进程。

坚持“规划先行、建管并重、统筹协调、科学发展”，以城乡融合发展核心区建设带动武威全域全面发展，着力打造新常态下全市经济增长的动力引擎。抓紧修编武威市城市总体规划、历史文化名城保护规划，编制完成凉州主城区南片、西北片、河东片控制性详细规划和道路交通、抗震防灾、绿地系统等专项规划，完善县城、园区和小城镇规划，推进新型城镇化试点县、镇“多规合一”等规划编制。科学构建城乡空间布局，凉古核心区要加快凉州城、古浪新区、大靖镇和园区、中小城镇、新农村建设，率先形成规模化的城镇和产业集群。推进民勤县域核心区“一心一带一片”、天祝县域核心经济区 “一心一川两翼”建设。加大城市建设力度，建成凉州新城区纬四路、滨河东路、滨河公园，建成投运污水处理厂污水再生利用项目。开工建设二环南路向东延伸道路和桥梁，连接南入市道路。开工建设天马湖景观治理三期工程。加快德促贷款建设城区集中供热项目，争取建成投运。推进钟楼巷历史文化街区建设。加大保障性安居工程建设和农村危房改造力度，加快推进新关村—荣华老厂区棚户区改造项目。全力实施民勤县和凉州区黄羊镇、古浪县黄羊川镇新型城镇化试点工作。推进新型农村社区和中心村建设，建立农村环境卫生综合整治长效机制，着力改善农村人居环境，创建省级“千村美丽”示范村10个、“万村整洁”村153个。

四、加大生态保护建设力度，巩固扩大生态文明建设成果。

围绕建设国家级生态安全屏障综合试验区，统筹抓好节水、造林、治沙、防污四个重点。扎实推进石羊河流域后期治理，强化水资源调度管理，大力开展人工增雨雪作业，确保平水年民勤蔡旗断面过水量达到2.9亿立方米以上、全市地下水开采量控制在4.84亿立方米以内。全面整改预评估中提出的问题，为国家正式验收做好准备。抓好祁连山水源涵养区工程。积极谋划石羊河流域后续治理项目。加大林业建设力度，抓好“三北”防护林、防沙治沙、退耕还林、石羊河流域防沙治沙及生态恢复等重点生态工程和红水河百里生态经济长廊等生态项目，新建甘蒙（宁）省界武威段480公里防沙治沙大林带和濒危野生动物民勤青土湖试验性放养基地。坚持机关干部义务压沙制度，开展大规模压沙造林活动。着力打造通道绿化精品工程。严格落实“五禁”规定，保护建设湿地，巩固生态治理成果。完成人工造林50万亩，封育36万亩。推进沙产业发展模式，继续推行工业治沙、养殖治沙新模式，科学开发利用和治理保护沙漠。加快发展沙漠特色养殖和林果、药材种植产业，建立复合经营、生态经济、移民安置相结合的现代沙产业发展模式和沙区新村镇，推进治沙林承包治理经营，拓展脱贫致富新领域。坚决治理环境污染，严格执行环境保护法，健全完善节能减排目标责任制，严格控制主要污染物排放，推进清洁生产，促进资源节约和综合利用。实施工业点源、农业面源和水质污染、大气污染、生活污染综合治理。实行污染物排放总量控制制度，禁止无证排污和超标准、超总量排污，实现污染治理全防全控，优化环境质量。

五、全面发展社会事业，着力保障和改善民生。

加快普及学前教育和高中阶段教育，启动武威一中迁建提升工程。大力发展现代职业教育，组建武威职教集团。全面完成武威职业学院一期扩建工程，确保通过国家骨干高职院校验收。推进标准化学校建设，全力促进县域义务教育均衡发展。积极推进医药卫生体制改革，落实好新农合支付方式改革、重大疾病新农合医疗保障、县级公立医院改革等任务。抓好城乡居民大病保险工作。扎实开展全省中医药工作先进市创建工作。建成投用武威重离子治疗肿瘤中心暨荣华颐养园、市人民医院整体搬迁、市中医院综合服务楼项目。全面落实计划生育各项政策措施，继续稳定低生育水平。大力发展文化体育事业，加快重点文化工程建设，建成市博物馆和影剧院，启动鸠摩罗什寺扩建项目。整合基层宣传文化、党员教育、科学普及、体育健身等设施，加快“乡村舞台”、综合文化服务中心建设。建成投用市体育馆，争取开工建设市体育健身广场、游泳馆、训练馆，完成8个乡镇及社区体育健身中心工程、100个村级体育场。落实更加积极的就业政策，搭建就业平台，推动实现更高质量就业。引导高校毕业生到基层、企业就业或自主创业，新增城镇就业2.2万人。加强全民国防教育、国防后备力量建设和人民防空工作，广泛开展“双拥模范城（县）”创建活动，推动军民融合发展。落实民族宗教政策，加快民族地区经济社会发展。重视青少年教育成长，发展妇女儿童事业。建立社会养老服务体系，发展老年服务业。推进扶残助残、社会救助和慈善事业发展。组织开展第三次全国经济普查，继续做好防震减灾、外事侨务、地方志、统计、档案、消防等工作。

在认真办好省列民生实事的基础上，集中力量办好10件实事：1.解决8万农村人口的饮水安全问题。2.新建改建农村公路700公里。3.实施棚户区改造6000户以上。4.实施农村危房改造、新房建设3万户，其中农村危房改造1.3万户。5.继续推进高深山区农牧民“下山入川”工程，完成移民1.75万人。6.实施贫困村整村推进项目50个。7.消除中小学校舍D级危房23万平方米。8.村干部报酬年补助标准增加4000元，达到1.6万元。9.建设2个县级医院住院楼和11个乡镇卫生院业务用房。10.提高城乡居民社会养老保险基础养老金、新型农村合作医疗和城镇居民基本医疗保险、城镇低保、农村低保、农村五保政府补助标准。

六、推动社会治理创新，维护社会和谐稳定。

坚持改革、发展、稳定三位一体不动摇，高度重视信访工作，规范信访事项受理办理程序，引导来访人依法逐级走访，深入推进访诉分离和网上信访信息系统建设，认真组织开展社会稳定风险评估，完善矛盾纠纷排查化解制度，把各种不稳定因素化解在基层和萌芽状态。创新社会服务管理工作新模式，以创建“平安县区、平安乡镇街道、平安农村社区”为重点，加快基层社会服务平台建设，加强流动人口和特殊人群服务管理，强化“下山入川”移民区和城乡融合新型社区社会管理服务。加强应急管理体系建设，提高综合防范和抵御灾害能力。抓好国家粮食收储供应安全保障工程。组织开展“食品药品安全质量规范年”活动，建成市食品检验检测中心项目，强化食品药品及产品质量安全监管，确保食品药品安全。加强价格监管，保持物价总水平基本稳定。严格落实安全生产主体责任和行业监管部门责任，深入开展安全生产专项行动，继续加强农村道路交通安全和农村消防安全管理，开展工业园区和劳动密集型企业火灾隐患专项整治，坚决治理各类事故隐患，严厉打击非法违法生产经营行为，杜绝重特大安全生产事故发生。认真落实支持藏区发展的政策措施，广泛开展民族团结进步创建活动，依法加强民族宗教事务管理，确保天祝藏区和谐稳定发展。加大社会治安重点地区和突出问题整治力度，健全完善社会治安防控体系建设。严厉打击暴力恐怖活动和宗教极端势力犯罪，严防暴恐分子潜藏渗透。

七、全面推进体制机制创新，开创改革开放新局面。

发挥经济体制改革牵引作用，积极稳妥地推进重点领域和关键环节改革。

着力推进创新驱动。加快省级创新型试点城市建设，完善产学研政相结合的技术创新体系，支持荣华等省级企业技术中心争创国家级企业技术中心，支持中国航天武威新能源装备制造产业园、甘肃凯帝斯电梯制造有限公司等重点企业申报省级企业技术中心，新认定省、市级企业技术中心和工程技术研究中心10个以上。支持企业争创著名商标、驰名商标，组建企业集团，提升企业核心竞争力。围绕装备制造、农产品加工、生物技术应用与开发等重点领域，筛选一批重大技术创新项目，争取国家和省上专项支持。推动我市企业与省内外高校和科研院所开展技术合作、技术交流和人才培养。

加快推进农业农村重点改革。加大农村土地承包经营权流转力度，大力培育新型农业生产经营主体，发展适度规模经营，规范提升农民专业合作组织。落实最严格水资源管理制度，健全完善水权分配、管理、保护和交易机制。抓好凉州区小型农田水利设施产权制度改革及创新运行管护方式试点，实施民勤县农业水价综合改革试点项目。深化集体林权制度配套改革，完成各类林业资源确权颁证，建立和完善集体林地、林木流转机制。

全力建设甘肃国际陆港。围绕打造丝绸之路经济带黄金节点，举全市之力建设甘肃国际陆港，带动发展外向型经济。完善武威保税物流中心服务功能，实现中欧国际货运班列“天马号”运营常态化。按照外向型、生态型、城乡融合型定位，尽快制定实施总体规划、专项规划和单体规划。布局建设以保税物流中心为核心的陆港中心区，构建黄羊、土门、泗水高铁、陆港现代农业四大组团，带动周边区域发展。加强口岸服务功能建设，依托“天马号”货运网络，形成特色优势产业的生产供应链和消费供应链，带动进出口加工、装备制造业、现代服务业集聚发展，构建以国际贸易、国际物流、金融服务及关联产业为支撑的陆港经济产业体系。加强与丝绸之路经济带沿线地区的合作交流，吸引进出口产品和加工贸易企业在我市集结，打造向西开放的国际物流平台和出口产品加工基地，促进拓展中亚、西亚和欧洲市场。

坚持“管好用活”土地。开展市县两级土地利用总体规划中期评估和调整完善工作，健全和落实耕地、基本农田保护制度。积极开展城乡建设用地增减挂钩试点工作，推进宅基地复垦工作，置换城镇建设用地指标。建立土地统筹使用机制，完善招拍挂制度，推进土地收储、整理和处置。落实最严格的集约节约用地制度，全力保障基础设施、民生工程、新兴产业等建设用地。鼓励支持市场主体规模开发利用、治理保护沙漠。

全面落实财税管理制度改革措施。健全部门预算标准体系，提高预算统筹能力。全面推进政府预决算、部门预决算及“三公”经费预决算信息公开，改进年度预算控制方式，建立跨年度预算平衡机制。清理规范财税优惠政策，稳步推进税制改革。加强财政资金拨付时效监管，提升资金使用效益。推进国库集中收付制度改革，严格执行公务卡结算制度。强化政府性债务管理，积极防范和化解债务风险。

创新投融资机制。整合改革发展集团，强化投融资功能。发行武威二期企业债，用好武威私募债。支持金融机构扩大金融服务覆盖面，规范发展小额贷款公司。加强银企对接，加强金融生态环境建设，加大信贷支持力度。大力推进农村产权确权颁证和抵押担保贷款工作，“双业”“双联”贷款重点支持设施农牧业提质增效和无棚贫困户发展设施农牧业。积极争取设立保险公司和民营银行。

八、精心谋划研究，切实做好“十三五”规划编制工作。

编制“十三五”规划是今年的一项重要工作。要全面把握面临的新形势、新任务和新要求，深化对市情的认识，深入开展调查研究，全面总结“十二五”规划实施的成效和经验，找准主要矛盾，抓住重大问题，积极谋划“十三五”时期发展思路、目标任务，重点研究谋划好新常态下对转型跨越发展带动性强的重大工程、重大项目和重大措施，加强与省上规划的衔接，争取使我市谋划确定的重大项目纳入省上“十三五”规划。强化上下联动和部门协调机制，创新规划编制方法，坚持开门编制规划，汇聚民智，凝聚共识，勾画未来五年我市经济社会发展的宏伟蓝图。

九、切实加强政府自身建设，努力建设法治政府。

深入学习贯彻党的十八大和十八届三中、四中全会精神，把依法行政贯穿于政府工作全过程，坚持为民、务实、清廉，加快建设法治政府、责任政府和服务政府。

全面推进依法行政。认真贯彻落实中央关于依法治国的决定和省委、市委关于依法治省、治市的实施意见，坚持法定职责必须为、法无授权不可为的原则，健全依法决策机制，把公众参与、专家论证、风险评估、合法性审查作为重大行政决策必经程序。坚持严格规范公正文明执法。全面推进政务公开，落实政府信息公开制度，清理并向社会公开各级政府及其工作部门行政权力清单。认真办理人大代表建议、政协委员提案，自觉接受人大法律监督和工作监督、政协民主监督及全社会舆论监督。

深入落实党风廉政建设主体责任。牢固树立不抓党风廉政建设就是严重失职的意识，始终把政府党风廉政建设作为重大政治任务摆在突出位置，认真履行主体责任、第一责任人责任和“一岗双责”，全面开展党风廉政建设主体责任约谈，直接传递责任，层层传导压力。严明政治纪律和政治规矩，深入落实中央八项规定和省委、市委“双十条”规定精神，完善制度机制，加强廉政教育和风险防控，扎紧制度“笼子”，强化对权力运行的制约和监督。加大对政策措施落实情况、民生项目和资金、工程建设、土地管理、公共资源交易的监督检查力度，依法治理和纠正损害群众利益的不正之风，严肃查处各类违纪违法案件，树立清正廉洁的良好形象。

加快政府职能转变。深化简政放权、放管结合改革，做好国务院、省政府取消和下放行政审批事项的承接落实，坚决杜绝审批事项权力变相截留，再精简下放一批行政审批事项。简化审批程序，提高效率。赋予凉州区县级人民政府土地管理权，下划城市建设、管理等职能，市级主要负责指导、规划、监督、考核工作。稳步推进事业单位分类改革。全面实行机构编制实名制管理，严格执行机构编制管理证制度。积极稳妥推进公车改革。

切实加强能力建设。按照好干部标准要求，坚持把加强干部队伍能力建设与全面深化改革、全面推进依法行政和全面建设小康社会结合起来，加快能力提升，养成持续学习知识、持续思考问题、持续研究工作、持续抓好落实的良好习惯。认真学习党的十八大、十八届三中、四中全会精神和习近平系列重要讲话精神，学习经济发展新常态理论，学习市场经济、金融、法律、科技等各方面的知识，既钻研宏观、又熟悉微观，既更新知识结构、又提升理论水平。立足落实省委、省政府和市委重大战略决策部署，深入开展调查研究，切实加强实践锻炼，坚持理论联系实际，既把握国情省情、又熟知市情，既解决实际问题、又不断增长实践才干，成为推动改革发展的行家里手。

坚持不懈改进作风。深化教育实践活动整改和专项治理工作，持续用力整治“四风”问题，强化制度约束，落实长效机制，巩固拓展活动成果。认真开展“工作落实年”活动，持之以恒地推进效能风暴行动，铁腕整治“为官不为”，严厉惩处“贪假懒庸”，集中精力解决工作落不实的顽症。特别要重视解决一些干部不敢担当、不愿负责，怕得罪人、当“太平官”，虚于应付、效率低下，只要不出事、宁愿不干事等问题。以踏石留印、抓铁有痕的决心，按照市委三届八次全委（扩大）会议暨全市经济工作会议的部署要求，全力推动各项工作落实。

各位代表，新的一年，新的征程。让我们紧密团结在以习近平同志为总书记的党中央周围，在省委、省政府和市委的坚强领导下，在市人大和市政协的监督支持下，紧紧依靠全市人民，改革创新，敢于担当，锐意进取，埋头苦干，为实现同全省一道全面建成小康社会而努力奋斗！
